# Sequencing and Functional Annotation of the Whole Genome of *Shiraia bambusicola*

**DOI:** 10.1534/g3.119.400694

**Published:** 2019-11-11

**Authors:** Xiyi Ren, Yongxiang Liu, Yumei Tan, Yonghui Huang, Zuoyi Liu, Xuanli Jiang

**Affiliations:** * College of Agriculture, Guizhou University, Guiyang, Guizhou, 550025, China,; † Institute of biotechnology, Guizhou Academy of Agricultural Sciences, Guiyang, Guizhou, 550006, China,; ‡ Guizhou Key Laboratory of Agricultural Biotechnology, Guiyang, Guizhou, 550006, China, and; § Guizhou Academy of Agricultural Sciences, Guiyang, Guizhou, 550006, China

**Keywords:** Genomic sequencing, Functional annotation, Phylogenetic analysis, Pathogenic gene, Secondary metabolism

## Abstract

*Shiraia bambusicola* is a rare medicinal fungus found in China that causes bamboo plants to decay and die with severe infection. Hypocrellin, its main active ingredient, is widely used in several fields, such as medicine, agriculture, and food industry. In this study, to clarify the genomic components, taxonomic status, pathogenic genes, secondary metabolite synthesis pathways, and regulatory mechanisms of *S. bambusicola*, whole-genome sequencing, assembly, and functional annotation were performed using high-throughput sequencing and bioinformatics approaches. It was observed that *S. bambusicola* has 33 Mb genome size, 48.89% GC content, 333 scaffolds, 2590 contigs, 10,703 genes, 82 tRNAs, and 21 rRNAs. The total length of the repeat sequence is 2,151,640 bp. The annotation of 5945 proteins was obtained from InterProScan hits based on the Gene Ontology database. Phylogenetic analysis showed that *S. bambusicola* belongs to Shiraiaceae, a new family of Pleosporales. It was speculated that there are more than two species or genus in Shiraiaceae. According to the annotation, 777 secreted proteins were associated with virulence or detoxification, including 777 predicted by the PHI database, 776 by the CAZY and Fungal Cytochrome*P*450 database, and 441 by the Proteases database. The 252 genes associated with the secondary metabolism of *S. bambusicola* were screened and enriched into 28 pathways, among which the terpenoids, staurosporine, aflatoxin, and folate synthesis pathways have not been reported in *S. bambusicola*. The *T1PKS* was the main gene cluster among the 28 secondary metabolite synthesis gene clusters in *S. bambusicola*. The analysis of the T3PKS gene cluster related to the synthesis of hypocrellin showed that there was some similarity between *S. bambusicola* and 10 other species of fungi; however, the similarity was very low wherein the highest similarity was 17%. The genomic information of *S. bambusicola* obtained in this study was valuable to understand its genetic function and pathogenicity. The genomic information revealed that several enzyme genes and secreted proteins might be related to their host interactions and pathogenicity. The annotation and analysis of its secondary metabolite synthesis genes and gene clusters will be an important reference for future studies on the biosynthesis and regulation mechanism of the secondary metabolites, contributing to the discovery of new metabolites and accelerating drug development and application.

## Background

*Shiraia bambusicola* is a type of fungus that is parasitic on a few bamboo tissues. It infects a few species of bamboo and is only distributed in Japan and southern China. Importantly, it is used in Chinese medicine for treating convulsions in children, rheumatoid arthritis, sciatica, lumbar muscle strain, bruises, cold stomach pain, and acute hepatitis (Jia *et al.* 2006; Li and Durbin 2009; Liu *et al.* 2011). More than 10 types of chemical substances have been isolated from *S. bambusicola*, including mannitol, stearic acid, hypocrellin A–D (HA, HB, HC and HD), hypomycin A, ergosterol, ergosterol peroxide, 1,8-2-hydroxyanthracene, 11,11′-dideoxyverticillin, *S. bambusicola* polysaccharides, and digestive enzymes (Wang *et al.*1990; Lin *et al.* 2002; Fang *et al.* 2006; Shen *et al.* 2002). Among these, hypocrellins are the most essential active substances; they are types perylenequinone compounds, which are used as natural coloring agents and novel photosensitizers in the food and medicine industries. Particularly, they have a very good effect on photosensitizing tumor cells, resisting viruses, treating retinopathy and diabetes, and inhibiting HIV-I virus proliferation. They have remarkable clinical effects against vulvar skin lesions in women, postburn scars, psoriasis, tinea capitis, tumors, and rheumatoid arthritis (Shao *et al.* 2011;Zhao *et al.* 2012). As antibacterial agents, hypocrellins have an obvious inhibitory effect on gram-positive bacteria, such as *Staphylococcus aureus*, and agricultural research has demonstrated that they have a certain inhibitory effect on fungi, such as rice sheath blight and some molds (Lu *et al.* 2012; Zhu *et al.* 2014). In addition, they have good potential as edible natural pigments because of their bright colors, good dyeing force, excellent fat solubility, and health-care functions (Shi *et al.* 2016). Moreover, the liver-protecting function of *S. bambusicola* polysaccharide has attracted much attention. However, several years of research has demonstrated that *S. bambusicola* does not satisfy the needs of clinical medication because of its low yield, difficulty in preservation and proliferation, strict hosts, and a great deal of plucking.

The taxonomic status of *S. bambusicola* has been controversial since 1900 when Hennings classified *Shiraia* into the Nectriaceae family of Pyrenecetes (Hennings 1900). In 1902, Saccardo attributed it to the Hypocreace family of Hypocreales because of its large fleshy fruit body (Saccardo 1902). In 1980, Amano indicated that the ascospores of *Shiraia* have a double-walled structure and thus, attributed it to the Polosporaceae family of Pleosporales and Loculoascomycetes (Amano 1980). However, because the hex-ascospore structure of *Shiraia* was inconsistent with the characteristics of the eight ascospores of the Pleosporaceae, Kirk *et al.* attributed it to the Dothideales of Loculoascomycetes in 2001 (Kirk 2001); but the status of the genus and family of *Shiraia* remained undetermined. It was found that there was only one species of the *Shiraia* genus, namely *Shiraia*. In 2004, Cheng *et al.* constructed a *Shiraia* phylogenetic tree based on 18S rDNA and ITS sequences, assigned it to Pleosporales, albeit with a different classification from that demonstrated in Amano’s study, and recommended *Shiraia* to be placed as an independent genus in the Phaeosphaeriaceae family (Cheng *et al.* 2004). In 2013, our research group officially established a new family—Shiraiaceae of the Pleosporales—based on the LSU-rDNA, EF, and RPB gene sequence data of *S. bambusicola* (Liu *et al.* 2013). To date, the family comprises only one genus and species.

For a long time, many questions related to the obligate parasite relationship between *S. bambusicola* and bamboo, the hypocrellin biosynthetic pathway, regulation mechanism, and gene information have remained unresolved. These questions have restricted the development and utilization of *S. bambusicola*. In this study, we sequenced and assembled the entire genome of *S. bambusicola* GZAAS2.1243 using the Illumina HiSeq 2000 platform (BGI-Shenzhen Co., Ltd.). Based on the genome analysis, we expected to identify the genes related to their pathogenicity, secondary 2metabolite biosynthesis, and growth and development on the host.

## Methods

### Strains, growth conditions, and genomic DNA

*S. bambusicola* GZAAS2.1243 was isolated from the Jing County, Anhui Province, China. The collected samples were kept at the Key Laboratory of Agricultural Biotechnology of Guizhou Province, Guiyang city, China. The fungus was cultured in PDA (Potato Dextrose Agar) medium (200g/L potatoextract, 20-g/Lglucose,18-g/L agar) plates in the dark at 25° for 7 days. Mycelia were collected in a mortar, liquid nitrogen was added, and the samples were crushed using a pestle. Genomic DNA from the fungal mycelia was extracted using the CTAB method. The DNA pellet was dissolved in sterile water and adjusted to a concentration of 552 μg/mL.

### Genomic DNA sequencing and assembly

#### Library Construction and Genome sequencing:

One paired-end library (insert sizes 270 bp) and one mate-pair library (5Kb) were constructed. The construction flow of library was as follows: 1 μg genomic DNA was randomly fragmented by Covaris. The fragmented DNAs were tested using Gel-Electrophotometer and purified using the AxyPrep Mag PCR clean up Kit. The fragmented DNAs were combined with End Repair Mix and incubated at 20° for 30 min. Subsequently, the end-repaired DNAs were purified using AxyPrep Mag PCR clean up Kit. The repaired DNAs were combined with A-Tailing Mix and incubated at 37° for 30 min. Illumina adaptors were ligated to the Adenylate 3′Ends DNA and incubated at 16° for 16 h. The adapter-ligated DNAs were purified with the AxyPrep Mag PCR clean up Kit and their fragments were then sorted and selected based on the insert size. Several rounds of PCR amplification with PCR Primer Cocktail and PCR Master Mix were performed to enrich the adapter-ligated DNA fragments. The PCR products were purified again using the AxyPrep Mag PCR clean up Kit. The library was qualified by the Agilent Technologies 2100 bioanalyzer and ABI StepOnePlus Real-Time PCR System. Paired-end sequencing was performed for the qualified libraries using Illumina System.

We used a whole-genome shotgun strategy and next-generation sequencing technologies on the Illumina HiSeq 2000 platform (BGI-Shenzhen Co., Ltd., Shenzhen city, Guangdongprovince, China) to sequence the *S. bambusicola GZAAS2.1243* genome.

#### Estimation of genome size using k-mer:

Based on the length of each k-mer as Kbp, a raw sequence read with L bp comprises (L – K + 1) k-mers. The frequency of each k-mer can be calculated from the genome sequence reads. k-mer frequencies along the sequence depth gradient follow a Poisson distribution in a given dataset, except for a higher representation of low frequencies owing to sequencing errors because these affect the number of k-mers that may be orphaned among all splitting k-mers. The genome size—G,—was defined as G = K_num/K_depth, where the K_num is the total number of k-mers and K_depth is the most occurring frequency.

#### Genome assembly:

The *S. bambusicola* genome was assembled *de novo* using SOAPdenovo 1.05 (http://soap.genomics.org.cn) (Li *et al.* 2010), which employs the de Bruijn graph algorithm to both simplify the assembly and reduce computational complexity. Low-quality reads were filtered out, and potential sequencing errors were removed or corrected by the k-mer frequency methodology. We filtered out the following type of reads: (1) reads having an “N” of >10% of its length, (2) reads from short insert size libraries having >65% bases with quality of ≤7 and the reads from large insert size libraries that contained >80% bases with quality of ≤7, (3) reads with >10 bp from the adapter sequence (allowing no >2 bp mismatches), (4) small insert size paired-end reads that overlapped ≥10 bp between the two ends, (5) read 1 and read 2 of two paired-end reads that were completely identical (and thus considered to be the products of PCR duplication), and (6) reads having a k-mer frequency of <4 after correction (to minimize the influence of sequencing errors).

After these quality control and filtering steps, the data were retained for assembly. SOAPdenovo first constructs the de Bruijn graph by splitting the reads from short insert size libraries (270 bp) into 41-mers and then merging the 41-mers; contigs, which exhibit unambiguous connections in de Bruijn graphs, are then collected. All reads were aligned onto the contigs for scaffold building using the paired-end information. This paired-end information was subsequently used to link contigs into scaffolds, step by step, from short insert sizes to long insert sizes. Some intra-scaffold gaps were filled by local assembly using the reads in a read pair, where one end was uniquely aligned to a contig, whereas the other end was located within a gap. To access assembly quality, high-quality reads that satisfied our filtering criteria were aligned onto the assembly using BWA with default parameter (Li and Gao *et al.* 2009). To test for completeness of the assembly, the sequencing depth of each base was calculated from the alignment, the proportion of a given depth was calculated, plotted, and compared with the theoretical Poisson distribution with a mean corresponding to the peak. If genomic regions collapse because of assembly quality, these regions would possess a higher than expected sequencing depth, *i.e.*, if two copies were merged into a single copy, the depth of the assembled region is expected to be twofold higher than the expected value.

### Genome annotation

#### Repeat annotation:

The repetitive sequences were predicted using the Repbase databases (https://www.girinst.org/repbase/), ProteinMask databases (http://www.Repeat-masker.org/cgi-bin/ RepeatProteinMaskRequest), TRF databases(http://tandem.bu.edu/trf/trf.html), *de novo* (download at https://github.com/Reedwarbler/REPdenovo) (Jiang *et al.* 2013; Chong *et al.* 2016). Tandem repeats were located across the genome using the software Tandem Repeats Finder (TRF) (http://tandem.bu.edu/trf/trf.html) (Benson 1999). Transposable elements (TEs) were predicted in the genome by homology to RepBase sequences using RepeatProteinMask (http://repeatmasker.org/cgibin/ Repeat Protein Mask Request) and RepeatMasker (http://www.repeatmasker.org/)with default parameters (Chen 2004).

#### Non-coding RNA annotation:

The tRNA region and tRNA secondary structure were predicted using a tRNA scan-SE online tool (http://lowelab.ucsc.edu/tRNAscan-SE/) with default parameters. The rRNA were predicted using *de novo* (Washietl 2010). The lncRNA and snRNA were obtained using the infernal software (http://eddylab.org/infernal/) with default parameters (LiangHu 2013).

#### Gene annotation:

To predict genes in the *S. bambusicola* genome, we used both homology-based methods and *de novo* methods. For the homology-based prediction, fungi proteins were downloaded from Ensembl (release 44) and mapped onto the genome using TblastN (Kent 2002). Subsequently, homologous genome sequences were aligned against the matching proteins using Genewise (Birney *et al.* 2004) to define gene models. For *de novo* prediction, Augustus (Stanke *et al.* 2003) and Genscan (Salamov *et al.* 2000) were employed to predict coding genes. Finally, the homology-based and *de novo*-derived gene sets were merged to form a comprehensive nonredundant reference gene set using GLEAN (http://sourceforge.net/projects/glean-gene/) by removing all the genes with sequences of <50 amino acid as well as those that only had *de novo* support. Genes were also annotated using Blast2GO (http://www.blast2go.com/b2ghome/) based on the terms “biological function,” “Cellular Component,” and “molecular process” in Gene Ontology (GO). To identify the genes involved in pathogenicity and virulence in the *S. bambusicola* genome, BLASTP with a cut-off E value set at 1e − 10 was adopted to search against the Pathogen Host Interactions database (PHI) (version 3.6) (http://www.phi-base.org/), which comprises experimentally validated pathogenicity, virulence, and effector genes of fungal, oomycete, and bacterial pathogens (Winnenburg *et al.* 2008). The genome-encoding cytochrome P450s were annotated using BLASTP to search the fungal Cytochrome P450 database (version1.1) (http://drnelson.utmem.edu/CytochromeP450.html) with a cut-off E value set at 1e − 10 (Moktali *et al.* 2012). Proteomes were classified into proteolytic enzyme families by performing a batch Blast search against the MEROPS protease database (release 9.13) (Rawlings *et al.* 2008), and carbohydrate-active enzymes were classified using a HMMER (v3.1b1, with default parameters) scan against the profiles compiled with dbCAN release 4.0 (Yin *et al.* 2012) based on the CAZY database (version 2012-03-13) (http://www.cazy.org/).

### Personalized bioinformatics analysis

Phylogenetic analysis: In this study, the information of the entire genome of *S. bambusicola* GZAAS2.1243 was phylogenetically analyzed for *S. bambusicola*, some species of Pleosporales, doubtful species of *S. bambusicola*, and some species of Ascomycotina (Supplemental Material, Table S1). Orthologous protein prediction was performed using Proteinortho (v5.11, with default parameters, except that identity =75) (Lechner *et al.* 2011). Among the predicted orthologous gene clusters, highly conserved single-copy gene clusters were chosen and aligned using the MUSCLE (v3.8.31_i86 linux64, with default parameters) (Edgar 2004) algorithm. To eliminate divergence and ambiguously aligned blocks from the alignment, Gblocks (v0.91b) was employed under the default parameter setting (Talavera *et al.* 2007). The trimmed alignments of orthologous sequences were concatenated using a Perl script FASconCAT (v1.02, with default parameters) (Kück *et al.* 2010), and a maximum likelihood phylogenetic tree was constructed using the Dayhoff model in TREE-PUZZLE v5.3.rc16 with 1000 bootstrap replicates (Schmidt *et al.* 2002). The tree was visualized using Figtree v1.42 (http://tree.bio.ed. ac.uk/ software/figtree).

Analysis of candidate genes associated with hypocrellins synthesis: (1) The gene set from 10 closely related species(Table S2) was applied for gene family analysis using the TreeFam software. Thereafter, the unique genes of *S. bambusicola* species were screened out. (2) To identify the common genes, gene prediction was conducted by gene sequence alignment among the above unique genes and genes of another species (*Shiraia* sp. slf14) that also produces hypocrellin. (3) Perylenequinone-producing species (a total of five species, one of which is transcriptome data) and perylenequinone-nonproducing species were used for gene family analysis (Table S3). Genes that did not appear in any perylenequinone- nonproducing species but could be detected in at least four perylenequinone-producing species were identified as target genes. The consensus genes were screened by comparing the target genes and the genes obtained from step (2).

On the homepage of the antiSMASH tool (https://antismash-db.Secondarymetabolites.org/#/start), the genomic sequence was entered for analysis of the secondary metabolite gene cluster with default parameters.

### Data availability

The whole-genome sequence data reported in this paper have been deposited in the Genome Warehouse in BIG Data Center, Beijing Institute of Genomics (BIG), Chinese Academy of Sciences, under accession number GWHAAZB00000000 and publicly accessible at http://bigd.big.ac.cn/gwh. Table S1 illustrates the phylogenomic analysis results of the species. Table S2 present the details of the 10 other species, which are closely related to *S. bambusicola* for gene family analysis. Table S3 reflects the perylenequinone-producing and perylenequinone-nonproducing species. Table S4 presents the summary of some main features of *S. bambusicola* and the 13 sequenced Pleosporales fungi genomes. Table S5 provides information on the secreted proteins. Table S6 presents the genes predicted in pathogen–host interactions. Table S7 presents the genes predicted in cytochrome P450. Table S8 presents the genes predicted in carbohydrate-active enzymes. Table S9 presents the genes predicted in protease. Table S10 presents the genes shared between protease and CAZY. Table S11 presents the genes shared among protease, CAZY, and PHI. Table S12 presents the genes shared between protease and P450. Table S13 presents the genes shared among protease, cytochrome P450, and CAZY. Table S14 presents the genes shared among protease, cytochrome P450, and PHI. Table S15 presents the genes shared among protease, cytochrome P450, CAZY, and PHI. Table S16 presents the genes shared between cytochrome P450 and CAZY. Table S17 presents the genes shared between CAZY and PHI. Table S18 presents the genes shared between cytochrome P450 and PHI. Table S19 presents the genes shared among cytochrome P450, CAZY, and PHI. Table S20 presents the genes shared between protease and PHI. Table S21 presents the shared genes related to the synthesis of hypocrellins between S. bambusicola and other species that produce hypocrellins. Table S22 presents the shared genes related to the synthesis of perylenequinones between the perylenequinone-producing and perylenequinone- nonproducing species. Table S23 presents the genes related to hypocrellins biosynthesis. Table S24 presents the genes cluster which related to hypocrellins biosynthesis. Supplemental material available at figshare: https://figshare.com/s/ffefab4fb2879489cb02.

## Results and analysis

### Genome sequencing and assembly

Genome sequencing of *S. bambusicola* was performed using the Illumina HiSeq 2000 platform, and the 3334 Mb raw sequence data were sequenced and filtered to obtain clean data. The *S. bambusicola* genome was assembled using the SOAPdenovo 1.05 software. The genome was assembled into 2590 contigs and 333 scaffolds with a combined length of 33,146,786 bp. The longest and shortest lengths of scaffolds were 1,295,965 and 1000 bp, respectively. The N50 and N90 values were 546,034 and 132,645 bp, respectively. The GC content was 48.89%. There were 262 fragments >2 kb (Table 1). The genome size of *S. bambusicola* was similar to that of most Pleosporales fungi, but the GC content was the least similar (Table S4).

**Table 1:** The statistical results of the genome assembly of *Shiraia bambusicola*

Sample Name	Contig	Scaffold
**Total Number (#)**	2,590	333
**Total Length (bp)**	30,897,795	33,146,786
**Total Number (≥100bp)**	2,590	333
**Total Number (≥2kb)**	1,986	262
**N50 (bp)**	28,267	546,034
**N90 (bp)**	1,922	132,645
**Max Length (bp)**	137,842	1,295,965
**Min Length (bp)**	200	1,000
**GC Content (%)**	48.89	48.89

### Genomic component

#### Gene prediction and annotation:

The composition of the *S. bambusicola* genome was estimated by gene prediction, repeat sequence prediction, and noncoding RNA prediction, and *S. bambusicola* genome was found to comprise 10,703 genes, 30,545 exons, and 19,842 introns. The total and average lengths of the gene were 18,123,210 and 1693.28 bp; exons, 15,921,153 and 521.24 bp; intron, 2,202,057 and 110.98 bp; and CDS region, 15,901,268 and 1485.68 bp, respectively (Table 2).

**Table 2:** The statistical results of genomic components of *Shiraia bambusicola*

	*Shiraia bambusicola*
**Genome Size (Mb)**	33.14
**Number of Gene**	10,703
**Number of Exons**	30,545
**Number of CDS**	10,703
**Number of Intron**	19,842
**Length of Gene (bp)**	18,123,210
**Length of Exons (bp)**	15,921,153
**Length of CDS (bp)**	15,901,268
**Length of Intron (bp)**	2,202,057
**Average Length of Gene (bp)**	1,693.28
**Average Length of Exons (bp)**	521.24
**Average Length of CDS (bp)**	1,485.68
**Average Length of Intron (bp)**	110.98

The coding regions accounted for 87.85% of the entire genome, and each gene contained about 2.85 exons and 1.85 introns. The total length of the repeats was 2,151,640 bp, which was close to 6.5% of the entire genome size.

### Repeat sequences

Genetic information-rich repetitive sequences were a part of the gene regulatory network, which could regulate the initiation of DNA replication and promote or terminate transcription with a variety of *cis*-regulatory elements and signal molecules(Yamamoto 2018). The repeat sequences included DNA transposons, tandem repeats(TR), and transposable elements, which were further divided into long terminal repeat transposons (LTR) and non-long terminal repeat transposons(Non-LTR), including long and short interspersed elements (LINE and SINE, respectively). The repetitive sequences were predicted using the methods of Repeat Masker, Repeat Protein Mask, Tandem Repeats Finder and *De novo*, and their total length was 2,151,640 bp, accounting for 6.4998% of the entire genome. Among them, the repetitive sequences were 551,548, 388,081, 506,412, and 1,566,152 bp based on the Repbase databases, ProteinMask databases, TRF databases, and *De novo* predictions, respectively, accounting for 1.6661%, 1.1723%, 1.5298%, and 4.73111% of the entire genome, respectively (Table 3). The prediction of different types of transposed elements revealed that the *S. bambusicola* genome comprised the following: DNA transposons of 887,263 bp, accounting for 2.6803% of the entire genome; LINE of 325,445 bp, accounting for 1.1428% of the entire genome; SINE of 2,417 bp; long terminal repeats of 316, 920 bp; others of 66 bp; and unknown of 3929 bp (Table 4).

**Table 3:** Repeat sequence statistics

Method	Repeat size (bp)	% in genome
Repbase	551,548	1.6661
ProteinMask	388,081	1.1723
Denovo	506,412	1.5298
TRF	1,566,152	4.7311
Total	3,012,193	9.0993

Description: Type, a method for predicting repeats; Repeat Size, the total length of repeats; % in Genome, percentage of repeats in the genome; Total, the deduplication total result of the four methods.

**Table 4:** Transposon classification information statistics

Type	Repbase TEs		ProteinMask TEs		Denovo		Combined TEs	
	Length (bp)	% in genome	Length (bp)	% in genome	Length (bp)	% in genome	Length (bp)	% in genome
**DNA**	212,137	0.6408	154,002	0.4652	212,137	0.6408	308,987	0.9334
**LINE**	90,148	0.2723	58,770	0.1775	90,148	0.2723	139,272	0.4207
**LTR**	242,851	0.7336	175,309	0.5296	242,851	0.7336	316,920	0.9574
**SINE**	2,417	0.0073	0	0.0000	2,417	0.0073	2,417	0.0073
**Other**	66	0.0002	0	0.0000	66	0.0002	66	0.0002
**Unknown**	3,929	0.0119	0	0.0000	3,929	0.0119	3,929	0.0119
**Total**	551,548	1.6661	388,081	1.1723	551,548	1.6661	726,355	2.1942

Description: Type, the type of transposon, namely: DNA transposon (DNA), long scattered repeat (LINE), long terminal repeat transposon (LTR), short scattered repeat (SINE), other types (Other), Unknown; Repbase TEs, results of transposon predicted using the Repbase database; ProteinMask TEs, prediction results with RepeatProteinMasker; *De novo*, prediction results using the *De novo* method; Combined TEs, the deduplication results of the three methods. Total, the comprehensive results of several types of transposons which remove redundancy.

### Noncoding RNAs (ncRNAs)

ncRNAs are types of functional RNA molecules found in bacteria, archaea, and eukaryotic organisms; they can perform biological functions in the form of RNA molecules, regulate gene expression at the gene transcription level, RNA maturation, and protein translation, and participate in all important physiological processes, such as development, differentiation, and metabolism (Mercer 2010; Taft *et al.* 2010; Expósitovillén 2018).

Analysis performed using the tRNAscan and Infernal software revealed that the *S. bambusicola* genome comprises four types of ncRNAs—21 rRNAs, 82 tRNAs, 2 lncRNAs, and 32 snRNAs. The total and average lengths of the tRNAs were 7689 and 93.76 bp, respectively, accounting for 0.0232% of the entire genome. The total and average lengths of the rRNAs were 8,840 and 420.95 bp, respectively, accounting for 0.0267% of the entire genome. The total and average lengths of the lncRNA were 485 and 242.5 bp, respectively, accounting for 0.0015% of the entire genome. The total and average lengths of the snRNAs were 2871 and 89.71 bp, respectively, accounting for 0.0087% of the entire genome (Table 5).

**Table 5:** Noncoding RNA statistics

Type	Copy number	Average length(bp)	Total length(bp)	% in genome
**tRNA**	82	93.76	7,689	0.0232
**rRNA**	21	420.95	8,840	0.0267
**lncRNA**	2	242.5	485	0.0015
**snRNA**	32	89.71	2,871	0.0087

Description: Type, the type of ncRNA; Copy number, the number of ncRNAs; Average length, the average length of ncRNA; Total length, the total length of ncRNA; % in the genome, the proportion of ncRNA in the genome.

### GO-based distribution of the genes

**Figure 1:**
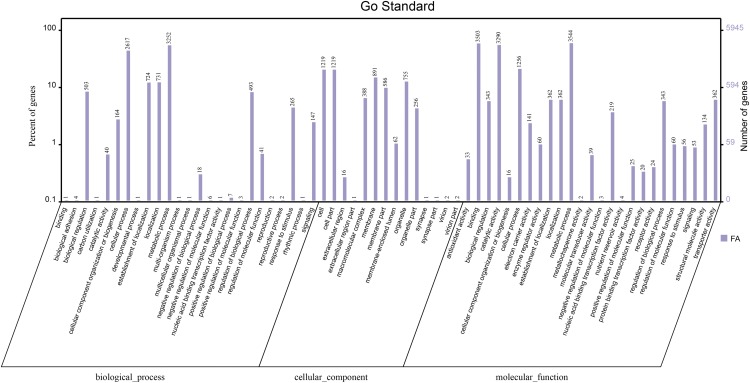
GO-based functional annotation of genes present in the [Shiraia bambusicola P. Hennings] genome. The first one indicates Biological Process domains, the second indicates cellular component domains, and the third indicates the Molecular function domains.

Among the total predicted genes in *S. bambusicola*, 5945 proteins (55.55%) had InterProScan hits; their genes were investigated for distribution into functional categories based on GO. According to the analysis using GO domains and Blast2GO (Conesa *et al.* 2005), Biological Process,” “Cellular Component,” and “Molecular Function” were the three generic terms of level data that are predicted (Figure 1). The major “Molecular Function” groups were established based on the following GO terms: “metabolic process” (59.61%), “binding” (58.92%), “catalytic activity” (55.34%), “establishment of localization” (6.09%), and “nucleic acid binding transcription factor activity” (3.68%). Among the cellular components, approximately 20.5% of the genes had involvements of the cell, followed by the membrane (14.99%), organelle (12.7%), virion (0.03%), synapse (0.02%). The “Biological Process” groups were established based on the following GO terms: “metabolic process” (54.7%), “cellular process” (44.02%), “localization” (12.3%), “biological regulation” (8.46%), and “response to stimulus” (4.46%).

### Phylogenetic relationships

To further confirm the taxonomic status of *S. bambusicola*, the information of the entire genome was phylogenetically analyzed for *S. bambusicola*, some species of Pleosporales, doubtful species of *S. bambusicola*, and some species of Ascomycotina in this study. The results showed that *S. bambusicola* belongs to Ascomycota, Leculoascomycetes, and Pleosporales. The three strains of *S. bambusicola* collected in this study were clustered and closely related to the *Stagonospora* sp. SRC1lsM3a of the Massarinaceae and *Parastagonospora nodorum* of the Phaeosphaeriaceae of Pleosporales, but *S. bambusicola* GZAAS2.1243 and the other two strains were not clustered. It was speculated that there are different genera or species in the Shiraiaceae; this speculation were different from the standpoints of previous researchers — only one species or one genus of the Shiraiaceae (Krik 2001; Liu *et al.* 2013).

### Predicted secreted proteins involved in virulence or detoxification

*Shiraia bambusicola* is a type of significant pathogenic fungus that can cause decay and death of bamboo plants. It was observed that the enzymes secreted by *S. bambusicola* played crucial roles in pathogenicity and virulence (Winnenburg *et al.* 2008; Lombard 2014). The secreted enzymes included PHI-related proteins, cytochrome P450 enzymes, carbohydrate-active enzymes, proteases, etc. With the *in silico* pipeline method, 777 of the 10,703 (7.26%) predicted proteins were potentially secreted proteins (Table S5), close to the average 8% found in most filamentous fungi (Han *et al.* 2016). By performing a whole proteome BLASTP against the pathogen–host interaction database, 1855 predicted proteins encoded by the *S. bambusicola* genome were determined to share homology with the genes implicated in pathogenicity and virulence, and 777 putative PHI-related proteins were potentially secreted proteins. Cytochrome P450 enzymes not only participated in the production of the metabolites important for the organism’s internal requirements but also played critical roles in adaptation to diverse environments by modifying harmful environmental chemicals. Using BLASTP to the fungal cytochrome P450 database, it was found that 900 of the 10,703 (8.4%) were cytochrome P450 protein (Table S7), and 776 putative cytochrome P450 proteins were potentially secreted proteins, most of which were involved in pathogen–host interactions. Using BLASTP to the Carbohydrate-Active Enzymes (CAZY) database, it was found that 1563 of the 10,703 (14.6%) proteins were predicted (Table S8) and 776 were potentially secreted proteins. A total of 493 proteases were secreted in the *S. bambusicola* genome (Table S9), and 441 were potentially secreted proteins.

**Figure 2:**
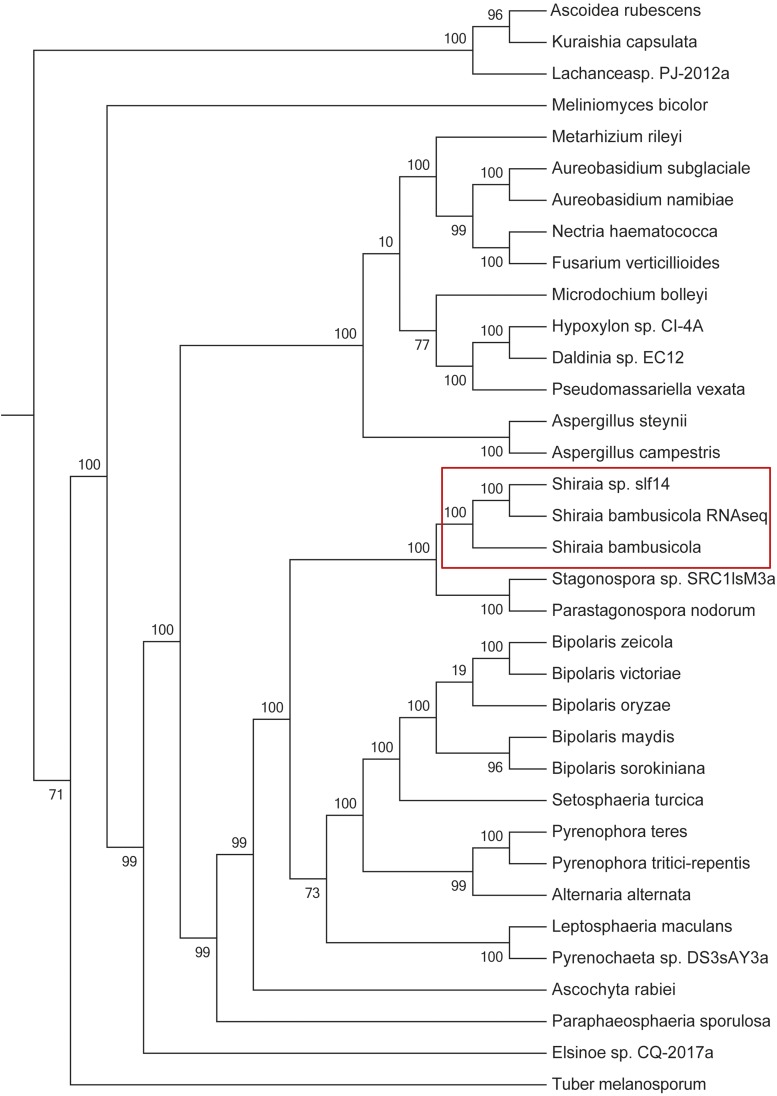
The phylogenetic relationship among the *Shiraia bambusicola* genomes, 33 other fungi with sequenced genomes, and one other *S. bambusicola* transcriptome.

**Figure 3:**
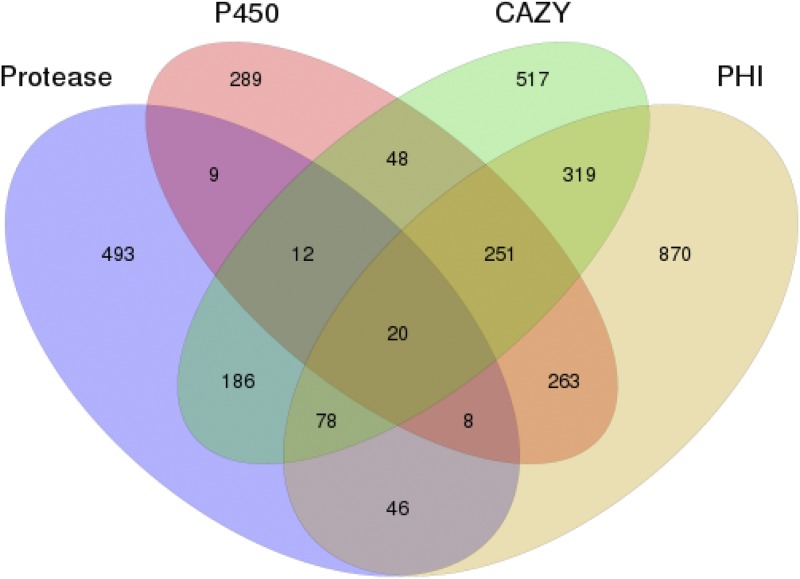
Venn graph showing the intersections among the proteases (blue), CYP450 enzymes (pink), CAZymes (green), and PHI proteins (light red).

The analysis of the four databases revealed the following: the PHI, cytochrome P450, proteases, and CAZY databases shared 440 secreted proteins; the cytochrome P450, CAZY, and PHI databases shared 776; the proteases, CAZY, and cytochrome P450 databases shared 440 (Table S10-S20 & Figure 2, 3).

### Potential pathogenesis-related genes

To the best of our knowledge, the genes/factors responsible for pathogenicity have not been determined in the *S. bambusicola* genome to date because of the lack of genomic resources. *Shiraia bambusicola* infects a bamboo plant, especially the leaf sheath of the branch for >2 years. To obtain the putative genes associated with pathogenicity, the proteomes of *S. bambusicola* were analyzed using the pathogen–host interaction gene database, cytochrome P450 enzymes, carbohydrate-active enzymes, secretory proteins, transporters, and transcription factors.

### Putative pathogen–host interaction genes

**Figure 4:**
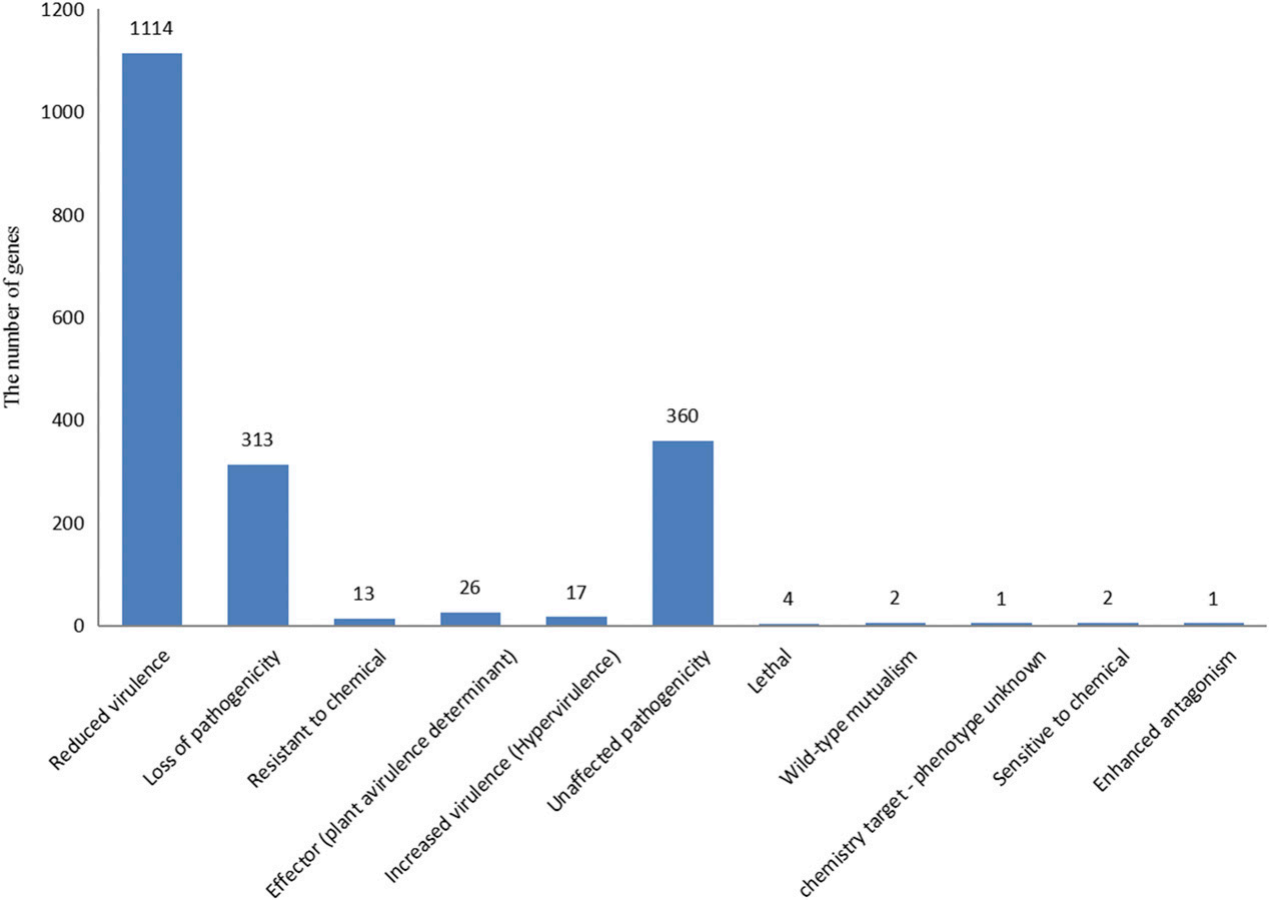
The gene distribution of pathogen-host interaction.

The PHI database contained the pathogenic, virulence, and effector genes validated by experiments on fungal, oomycete, and bacterial pathogens of fungi, insects, plants, and animals (Winnenburg 2008). To find the potential pathogenesis-related genes, the entire genome was annotated with a pathogen**–**host interaction database (gene database version 3.6 at E < 1 × 10^−20^). All the 10,703 protein sequences of *S. bambusicola* were aligned to PHI fungal genes using BLASTP (E value of 10^−10^). All 1855 (17.31% of the total genes of *S.bambusicola*) putative PHI genes were identified, and they spanned across 47 fungal species (Table S6).The highest number of homologs was found in *Magnaporthe grisea* (311 genes), followed by *Botrytis cinerea* (275 genes), *Candida albicans* (262 genes), *Cryptococcus neoformans* (124 genes), *Ustilago maydis* (113 genes), *Fusarium oxysporum* (91 genes), *Stagonospora nodorum* (69 genes), *Colletotrichum lagenarium* (68 genes), *Cercospora nicotianae* (62 genes),*Aspergillus fumigatus* (51 genes), and other fungal species (427 genes). These genes perform different functions in the process of pathogen–host interaction. Most of them are involved in reduced virulence (1114 genes), unaffected pathogenicity (360 genes), loss of pathogenicity (313 genes), effector (plant avirulence determinant; 26 genes), increased virulence (hypervirulence; 17 genes), resistance to chemical (13 genes), lethality (4 genes), wild-type mutualism (2 genes), sensitivity to chemicals (2 genes), chemistry target-phenotype unknown (1 gene), and enhanced antagonism (1 gene) (Figure 4).

### Carbohydrate-active enzymes

Carbohydrate-active enzymes are important for the pathogenicity and toxicity of fungus and are responsible for breaking down the cell wall components of the host to establish a successful infection process. They help the pathogens acquire nutrients by degrading the storage compounds in the host (Lombard 2013). The analysis of the CAZY database revealed that there are 1546 genes encoding carbohydrate-active enzymes, which fall into 153 CAZY protein families (Table S8). Among these genes, 206 proteins have transmembrane domains and 35 have signal peptides. Based on the analysis of catalytic activity using the CAZY database, it was observed that there are 677 genes that encode glycoside hydrolases (GH), which are distributed in 68 families and involved in the hydrolysis of the glycosidic bond between or within carbohydrate molecules. There are 381 glycosyltransferase (GT) genes, which were distributed across 39 families, and they use dolichol-P-glucose, polyprenol-P -mannose, and CMP-β-*N*-acetylneuraminate as the sugar donor. There are332 genes encoding carbohydrate-binding module (CBM) proteins, which belong to 25 families, and their main function is to bind cellulose, granular starch, and other substrates. The carbohydrate esterases (CE) are encoded by 179 genes, and they belonged to 14 protein families. There are 14 polysaccharide lyase (PL) enzyme genes assigned to three protein families (Table 6).

**Table 6:** Results of classification and annotation of carbohydrate enzymes

Classification	Number of CAZymes proteins	With transmembrane domain	With signal peptide	Number offamilies
**CBMs**	322	53	8	25
**CEs**	179	25	4	14
**GHs**	667	106	14	68
**GTs**	381	17	8	39
**PLs**	14	5	1	3

Description: CBM, carbohydrate-binding module; CE, carbohydrate esterases; GH, glycoside hydrolases; GT, glycosyl transferase; PL, polysaccharide lyase.

### Cytochrome P450 enzymes

Cytochrome*P*450(CYP) is a monooxygenase superfamily that performs several functions in fungi. These enzymes play important roles in the secretion and transport of fungal toxins, and regulate fungal pathogenicity and life cycle. A total of 900 cytochrome *P*450 genes were predicted, which are distributed across 52 gene families and 58 fungal species(Table S7).

### Secondary metabolites

**Figure 5:**
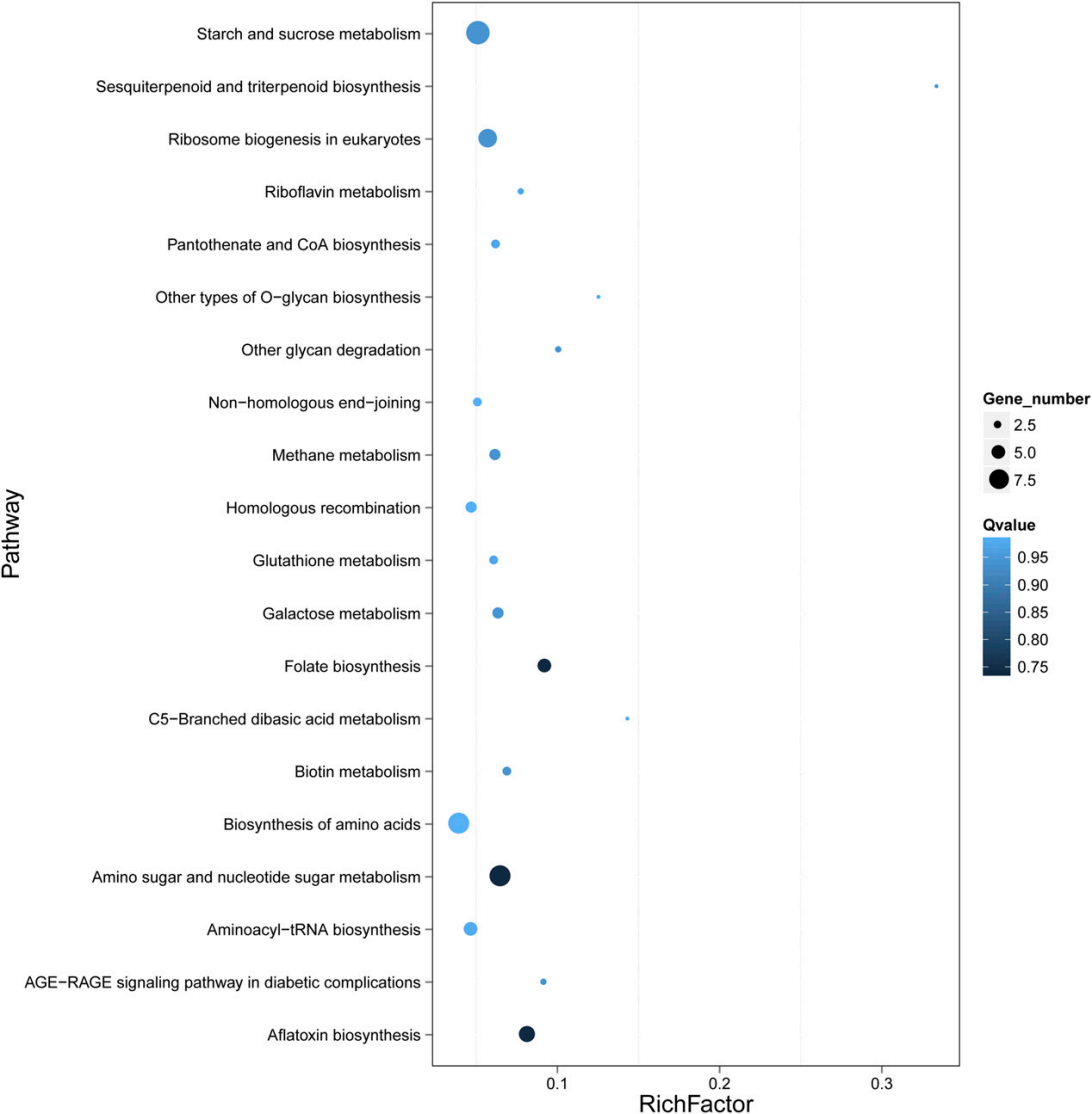
The partial results of the secondary metabolites pathway enrichment. The size of the black dot indicates the number of genes; the larger the dot, the more the genes are enriched. The blue shade indicates the significance of enrichment: the lighter the color, the closer it is to 1, the more significant is the enrichment.

The filamentous fungi produce various mycotoxins or other bioactive compounds that have long been exploited by the pharmaceutical industry (Khaldi *et al.* 2010; Medema *et al.* 2011). In China, *S. bambusicola* is used as a rare and traditional medicine. There are various medicinal active ingredients (secondary metabolites) in *S. bambusicola*; among these, hypocrellins are the most important. Hypocrellins are photosensitive compounds and perylenequinoid–type pigments with good application prospects and developing values in phototherapy, photodynamic pesticide, food additive, cosmetic pigment, and photoelectric conversion material. To determine the genes associated with the production of hypocrellins in *S. bambusicola* GZAAS2.1243, gene family analysis was performed using a gene set of 10 closely related species based on TreeFam software. Approximately 1353 unique genes were identified and applied to predict hypocrellin–producing genes by alignment to the genes from *Shiraia* sp. Slf14 (a hypocrellin production species). Eventually, 719 hypocrellin–producing genes (Table S21) were obtained. Both species that produce and do not produce perylenequinones were included in the gene family analysis. Initially, 395 perylenequinone–producing genes (Table S22) were screened. Based on the preliminary screening results and criteria, 251 shared genes (Table S23) were screened between the 395 perylenequinone–producing genes and the 719 hypocrellin–producing genes. All the 251 shared genes were enriched into 88 pathways using the pathway enrichment analysis (Table S23). There are > five genes for 28 pathways, and the three main enriching pathways were: and the three main enriching. The partial results of the pathway enrichment are shown in Figure 5. The synthetic pathway genes of terpenoids, staurosporine, aflatoxin, and folate have not been reported in *S. bambusicola* in the enriched pathway.

**Figure 6:**
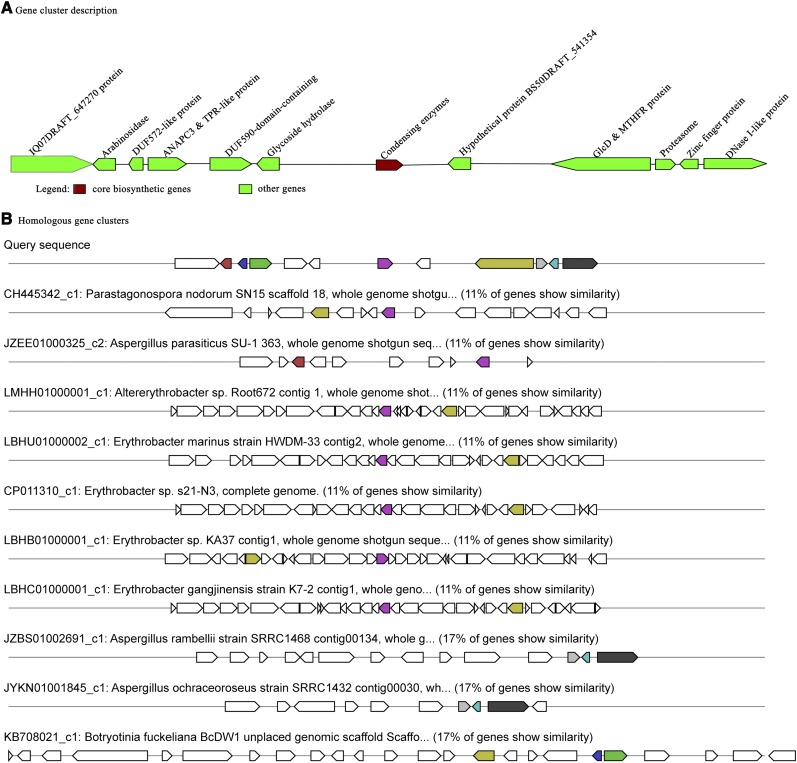
T3PKS gene cluster analysis.

The secondary metabolite gene clusters were analyzed using the antiSMASH tool. A total of 28 gene clusters of secondary metabolites were found (Table S24). Among them were 14 T1PKS, 1 T3PKS, 4 NRPS, 4 terpenes, 3 T1PKS-nrps, and 2 undefined gene clusters (Table 7). One T3PKS gene cluster, which is the key synthase in the hypocrellin synthetic process, was obtained in *S. bambusicola* (Zhao *et al.* 2016). T3PKS, representing chalcone synthase in plants, is a PKS similar to phenyl styrene ketone synthase discovered by Funa *et al.* 2007. This synthase directly catalyzes the condensation of pancosyl-CoA without ACP and is mainly responsible for the biosynthesis of monocyclic or bicyclic aromatic polyketides (Peng *et al.* 2015). A total of 17 genes and 12 CDS were predicted in the T3PKS gene clusters using the antiSMASH tool. The T3PKS gene clusters were located on scaffold 1 (locus: 553639–596757). and successively encoded 12 proteins (Figure 6A).

**Table 7:** The secondary metabolite synthesis gene clusters in *Shiraia bambusicola*

Type	Count
T1PKS	14
T3PKS	1
NRPS	4
Terpene	4
T1pks-nrps	2
undefined	3
Total	28

Gene cluster analysis revealed that the T3PKS gene clusters of 10 species were similar to that of *S. bambusicola*; however, the similarities were poor. The T3PKS gene cluster revealed that 11% of the gene is similar to *S. bambusicola* and other 7 species, and 17% of the genes show a similarity between *S. bambusicola* and other 3 species. Among them, the condensing enzymes and GlcD- and MTHFR-coding regions of *S. bambusicola T3PKS* were similar to those of *P. nodorum*, *Altererythrobacter* sp., the *E. marinus* strain HWDM-33, *Erythrobacter* sp. s21-N3, *Erythrobacter* sp. KA37, and the *E. gangjinensis* strain K7-2. The condensing enzymes and arabinosidase-coding regions of the *S. bambusicola T3PKS* were similar to those of *A. parasiticus* SU-1 363. The proteasome, zinc finger protein, and DNase I-like protein-coding regions were similar between *S. bambusicola* and the *A. rambellii* strain SRRC1468 and *A. ochraceoroseus* strain SRRC1432. The DUF572-like protein, ANAPC3, TPR-like proteins, and GlcD- and MTHFR-coding regions of *S. bambusicola T3PKS* and *B. fuckeliana* BcDW1 unplaced genome were similar (Figure 6 B).

## Discussion

*Shiraia bambusicola* is a cherished and endangered medicinal fungus and also a pathogen causing bamboo red dumpling disease. The entire genome of *S. bambusicola* GZAAS2.1243 was sequenced, assembled, and analyzed in this study. The *S. bambusicola* genome was estimated to be of 33 Mb, which is similar to the genome size of most of the sequenced strains of Pleosporales. The composition of this genome, including gene numbers, exons, introns, repeats, ncRNAs, and transposons, was revealed through interpretation of the assembly data. Data related to its genetic constituent were systematically analyzed. The classification status was confirmed based on the genomic information and was found to be identical to the findings of our previous study. *Shiraia bambusicola* GZAAS2.1243, *Shiraia* sp. slf14, and *S. bambusicola* RNA Seq were clustered to the same branch and were close to other strains of Pleosporales. It was speculated that there is more than one genus or one species in Shiraiaceae. This conclusion was different from the standpoints of previous researchers — only one species or one genus of the Shiraiaceae (Krik 2001; Liu *et al.* 2013). Among the total 1855 pathogen–host interaction genes in *S. bambusicola*, 777 proteins were potentially secreted proteins. The reduced virulence-related protein was the most in number, followed by unaffected pathogenicity and loss of pathogenicity. Among the 1563 carbohydrate enzymes, the most common was GH enzymes, followed by GT and CBM enzymes. The number of cytochrome *P*450 enzymes and proteases was 900 and 493, respectively.

It was gradually realized that *S. bambusicola* has a high application development value with deep research. The fruiting body could be immersed in wine and used as medicine; moreover, various medicinal components could be extracted from its fruiting body or culture. In this study, 252 *S. bambusicola*-specific genes were obtained by screening the secondary metabolism-related genes and enriched into 88 pathways. Among them, to the best of our knowledge, the terpenoids, staurosporine, aflatoxin, and folate synthesis pathways remain unreported to date. The terpenoids have many physiological activities, such as expectorant, relieving cough, dispelling wind, diaphoresis, deworming, and acting as an analgesic. Staurosporine is a typical ATP-competitive kinase inhibitor and a potential anticancer drug. Folate plays an essential role in the utilization of sugar and amino acids by *S. bambusicola* and is required for cell growth and reproduction. This study provides supporting evidence for the analgesic, wind-dispelling, and anticancer effects of terpenoids, which have previously been reported. It was speculated that terpenoids, staurosporine, and folate are synthesized in *S. bambusicola*.

The researchers have always been concerned about the medicinal value of *S. bambusicola*. Previously, to the best of our knowledge, there were no reports describing whether *S. bambusicola* is poisonous. In this study, it was found that there are six genes enriched for the aflatoxin synthesis pathway. These genes are involved in the regulation of aflatoxin synthesis; however, whether aflatoxin is synthesized in *S. bambusicola* needs to be confirmed by further experiments. A total of 28 secondary metabolite synthesis gene clusters were predicted using the antiSMASH tool. It is noteworthy that the T3PKS gene clusters might comprise the key enzymes of hypocrellin synthesis (Zhao *et al.* 2016) in *S. bambusicola* GZAAS2.1243. This gene cluster comprises 12 coding regions, and its core coding region is a condensing enzyme superfamily. The T3PKS gene cluster is similar to that of 10 other species, but the similarities are low. There is a high similarity of the *T3PKS* gene cluster between *S. bambusicola* and the *A. rambellii* strain SRRC1468, the *A. ochraceoroseus* strain SRRC1432, and *B. fuckeliana* BcDW1; however, this similarity is only 17%.

Genomic data of the *S. bambusicola* GZAAS2.1243 strain were systematically and comprehensively analyzed. The taxonomic status was verified based on the genomic information for the first time. To determine the pathogenic and secondary metabolite synthesis mechanisms, the related genome information was analyzed. We believe that this study will be a reference for exploring the *S. bambusicola*–host interaction genes. Further, this study establishes the foundation for future studies on the infection mechanism between *S. bambusicola* and the host. This study is expected to provide a basis to find the biosynthesis and the regulation genes’ information on secondary metabolism. We believe that this study will help accelerate the interpretation of the genetic basis of *S. bambusicola*.
